# AI and speech-based identification of cognitive impairment in maintenance haemodialysis—the ASPIRE study

**DOI:** 10.1093/ckj/sfag316

**Published:** 2026-09-11

**Authors:** Anne Møller Lohmann, Maria Dolmer Knop, Laurine Dargaud, Abhista Partal, Dimitrios Salogiannis, Henrik Birn, Jeanette Finderup

**Affiliations:** Department of Renal Medicine, Aarhus University Hospital, Aarhus, Denmark; Department of Clinical Medicine, Aarhus University, Aarhus, Denmark; Office of Health and Partnership, Vejle Hospital, Vejle, Denmark; Technical University of Denmark, Lyngby, Denmark; Technical University of Denmark, Lyngby, Denmark; Technical University of Denmark, Lyngby, Denmark; Department of Renal Medicine, Aarhus University Hospital, Aarhus, Denmark; Department of Clinical Medicine, Aarhus University, Aarhus, Denmark; Department of Renal Medicine, Aarhus University Hospital, Aarhus, Denmark; Department of Clinical Medicine, Aarhus University, Aarhus, Denmark

**Keywords:** AI, chronic kidney disease, cognitive impairment, dialysis, language

## Abstract

**Background:**

Cognitive impairment (CI) is common among individuals receiving maintenance haemodialysis and is associated with impaired treatment adherence, reduced quality of life, and mortality. However, CI often remains under-recognized in nephrology care because existing diagnostic approaches are resource-intensive. Speech-based artificial intelligence (AI) is a promising approach for identifying CI through analysis of speech characteristics. This proof-of-concept study investigated whether a speech-based AI model could identify Montreal Cognitive Assessment (MoCA)-based CI and distinguish individuals receiving haemodialysis from clinically relevant controls.

**Methods:**

Adults receiving maintenance haemodialysis and controls completed the MoCA and speech tasks. Participants were categorized into low-, moderate-, and high-risk CI groups according to MoCA scores. Acoustic, linguistic, and multimodal speech features were extracted using machine-learning models. Model performance was evaluated.

**Results:**

Speech-based AI differentiated individuals receiving haemodialysis from controls (*n* = 40). Multimodal models achieved the highest performance, with accuracies ranging from 0.78 to 0.80 for uraemia classification. For MoCA-based cognitive risk classification (*n* = 63), verbal fluency tasks yielded the strongest results. The best-performing model was the two-task fusion, which identified the high–risk group with a weighted F1–score of 73%, area under the receiver operating characteristic curve (ROC AUC) of 79%, and Matthew’s correlation coefficient (MCC) of 0.43. Moderate MoCA-based cognitive risk was more difficult to classify.

**Conclusion:**

Speech–based AI suggested potential as a scalable, non-invasive, and low-burden approach for identifying CI. Verbal fluency tasks and multimodal speech analysis showed the strongest classification performance. Although larger multicentre studies are needed, these findings provide proof of concept that speech may serve as a clinically relevant biomarker of CI in nephrology and support further validation using comprehensive neuropsychological assessment as a reference standard.

KEY LEARNING POINTS **What was known:**

- Cognitive impairment is highly prevalent in individuals with chronic kidney disease, particularly those receiving haemodialysis, and is associated with increased morbidity, mortality, and reduced quality of life, yet remains underdiagnosed in routine clinical care.
- Standard screening tools such as the Montreal Cognitive Assessment (MoCA) are resource-intensive, require trained personnel, and lack universally validated cut-offs in chronic kidney disease populations, limiting their scalability and clinical applicability.
- Speech-based AI has shown promise for detecting cognitive impairment in neurological populations, but has been minimally explored in nephrology populations.

**This study adds:**

- Speech-based AI was able to identify individuals at high risk of cognitive impairment among people receiving maintenance haemodialysis.
- Multimodal models combining acoustic and linguistic speech features achieved the strongest overall classification performance.
- Verbal fluency tasks showed the greatest potential for identifying MoCA-based cognitive risk from brief speech recordings.

**Potential impact:**

- Speech-based AI may provide a scalable, low-burden approach to cognitive screening in nephrology care.
- Earlier identification of cognitive impairment could support more personalized communication, treatment planning, and patient support.
- These findings provide proof of concept for larger validation studies using comprehensive neuropsychological assessment as the reference standard.

## BACKGROUND

Cognitive impairment (CI) is a prevalent complication in individuals with chronic kidney disease (CKD). The prevalence of CI increases with the progression of CKD, ranging from ~16%–38% in individuals with advanced CKD (grades 4 and 5) to 49.1%–53% among those receiving maintenance haemodialysis [1–3].

This cognitive decline is driven by structural and functional brain changes associated with CKD, including the accumulation of uremic toxins, neuroinflammation, and cerebrovascular dysfunction, which collectively impair neural integrity and cognitive processing [1, 4]. These impairments affect multiple domains, including attention, memory, and executive function, and can, among other things, compromise patients’ ability to manage complex treatment regimens, adhere to dietary and fluid intake, and participate meaningfully in shared decision-making [4, 5]. Therefore, CI in individuals with CKD is associated with increased morbidity and mortality, and reduced quality of life [2, 3, 6–8].

Early identification of CI is essential to ensure appropriate treatment planning [9, 10]. However, there is a lack of standard recommendations and consensus for the screening and diagnosis of CI in CKD, and standardized tools, such as the Mini-Mental State Examination and Montreal Cognitive Assessment (MoCA), have no universally validated cut-off scores for this population [3, 11]. MoCA is generally more sensitive to mild CI and includes an assessment of executive function, which is particularly significant for this population [12]. However, the gold standard would still be a comprehensive battery of neuropsychological tests [3].

Recent advances in artificial intelligence (AI) offer promising alternatives, with machine-learning methods analysing speech and language showing diagnostic potential in healthcare [13, 14]. Analyses of connected speech, particularly in picture description tasks, have demonstrated utility in detecting CI and Alzheimer’s disease [15]. Affected individuals often exhibit subtle changes such as reduced lexical diversity, increased pausing, and impaired syntactic complexity, reflecting underlying cognitive deficits. Unlike structured tests, connected speech captures natural language use and may reveal impairments overlooked by brief screening tools [16]. This approach is particularly relevant in CKD populations, where executive and language-related deficits are common but underassessed, and may therefore be adaptable to nephrology settings [15].

This proof-of-concept study aims to generate preliminary evidence for the performance of a speech-based AI model in a population receiving maintenance haemodialysis (uraemia) or with hypertension by examining two classification tasks. Detection of CI from structured speech recordings is clinically motivated, as it may support clinical management. By contrast, detection of uraemia from speech is investigated as an exploratory objective to assess whether speech features capture uraemic changes and to investigate whether uraemic CI differs from CI more broadly. The study further explores key methodological, technical, and clinical considerations relevant to assessing the model’s diagnostic accuracy, potential biases, and applicability within healthcare settings.

## MATERIALS AND METHODS

### Study design

This was a single-centre diagnostic case-control study conducted at the Department of Renal Medicine at Aarhus University Hospital, Denmark. Participants were recruited from two outpatient clinics within the department: (1) the dialysis clinic and (2) the hypertension clinic. The eligibility criteria aimed to ensure that all participants could complete the speech-based cognitive assessment. Inclusion criteria for both groups were age >18 years of age, fluent in Danish, and completion of at least seven years of formal education. Dialysis patients were further required to have been receiving maintenance haemodialysis for a minimum of 3 months, while patients with hypertension were required to have an estimated glomerular filtration rate >45 ml/min/1.73 m². Exclusion criteria included diagnosed dementia, significant speech impairments, and sensory deficits affecting hearing or vision.

Dialysis patients were approached during treatment sessions, and speech recordings were collected within the first 30 min of dialysis to minimize the influence of possible treatment-related cognitive fluctuations [17, 18]. Patients without kidney failure were recruited and recorded immediately following their first clinical consultation at the hypertension clinic.

Models were developed and evaluated for two predefined tasks: uraemia classification (distinguishing individuals with and without uraemia) and MoCA-based cognitive risk classification (identifying cognitive risk across all participants). In the uraemia task, cases were dialysis patients and controls had no kidney failure. In the cognitive task, participants were grouped into three risk categories based on MoCA scores.

Findings were reported in accordance with the Strengthening the Reporting of Observational Studies in Epidemiology (STROBE) guidelines for case-control studies [19].

### Cognitive test and speech recording

#### The Montreal cognitive assessment

Cognitive function was assessed using the MoCA, a validated screening tool for CI with high sensitivity and specificity in haemodialysis populations [9, 11, 20]. The total score ranges from 0 to 30, with higher scores indicating better cognitive performance. CI was stratified into three risk categories reflecting the absence of universally accepted MoCA cut-off values in CKD populations and to capture gradations of cognitive decline (MoCA score ≥26 low risk; 23–25 moderate risk; ≤22 high risk). This categorization also enabled multi-class classification modelling and allowed the AI model to distinguish between different levels of impairment rather than a binary outcome. All MoCA assessments were administered in paper format by two certified researchers using standardized protocols to minimize bias [21]. To reduce distractions, portable partitions were used in the dialysis clinic and private consultation rooms in the hypertension clinic.

#### Speech recording

Following MoCA testing, speech samples were collected using recorders. Participants completed a structured set of language tasks designed to capture multiple cognitive domains (see Table 1) [22]. Following preliminary cross-validation analyses, speech tasks 1–4 did not yield sufficiently informative features for the uraemia classification task (see File S1) and were therefore excluded from uraemia classification model development. Consequently, uraemia classification analyses were based solely on speech tasks 5 and 6. Because task selection was informed by model performance, results should be interpreted as exploratory. MoCA-based cognitive risk classification analyses were conducted separately and included all six speech tasks.

**Table 1:** Speech tasks.

1	Naming as many animals as possible in one minute
2	Generating words beginning with the letter ‘S’ in one minute
3	Generating words beginning with the letter ‘F’ in one minute
4	Explaining how to prepare an open-faced cheese sandwich
5	Describing a picture (‘the cookie theft’)
6	Retelling the Cinderella story using pictures from the book (or creating a structured narrative if unfamiliar with the story)

### Data processing and statistical analysis

#### Training of the speech-based AI model

Speech recordings were used to train and evaluate supervised machine-learning classification models [23]. Speech recordings underwent preprocessing with DeepFilterNet to remove background noise while preserving speech quality [24]. To minimize the impact of class imbalance on classification performance, balanced datasets were used for model training and evaluation. Denoising and balancing were performed before cross-validation, while feature scaling was performed within each fold to minimize information leakage. These procedures were incorporated into the final analytical pipeline to improve model robustness and generalizability.

For model training, speech recordings were labelled according to participants’ status for supervised learning across both classification tasks. For the uraemia classification task, a machine-learning approach was employed, incorporating both acoustic features and linguistic features to capture complex, non-linear relationships between speech and uraemia status. Two non-uraemia participants originally reserved for a hold-out test set were not included in the final balanced training dataset. All remaining non-uraemia participants (*n* = 20) were included, and uraemia participants were selected by exact matching on sex and closest similarity in age and MoCA score, resulting in a balanced dataset of 20 cases and 20 controls. No participant duplication, oversampling, or synthetic data generation was performed. No speech-derived features or model outputs were used during matching. For the MoCA-based cognitive risk classification task, models were developed using linguistic speech features only. Model development followed a feature-driven approach, focusing on linguistic markers rather than fully automated deep learning. Both classification tasks’ models were trained with a 10-fold cross-validation. Deep-learning hyperparameters were optimized using the same cross-validation procedure employed for performance estimation, whereas conventional machine-learning models were fitted using default hyperparameters. No nested cross-validation framework was used.

#### AI features

A range of supervised machine-learning models was applied to capture different patterns in the speech-derived features (see File S2). These included probabilistic, distance-based, and ensemble methods to ensure robustness across feature types. Feature sets included acoustic features, linguistic features, and combined multimodal representations. No performance-based feature selection was applied, and models were fitted using the complete set of extracted features or embeddings.

#### Variables

##### Uraemia classification task

The primary outcome variable was case–control status. Predictor variables included speech-derived embeddings extracted from pre-trained language and speech recognition models. The analysis subset was selected to achieve balance between cases and controls with respect to sex, age, and cognitive performance, as described above.

##### MoCA-based cognitive risk classification task

The primary outcome variable was MoCA-based cognitive risk status, categorized into three groups (see section: The Montreal cognitive assessment). Predictor variables were speech-derived features extracted from acoustic and linguistic analyses.

#### Study size

Sample size was determined based on feasibility and prior studies assessing CI in patients on haemodialysis. No formal power calculation was performed due to the exploratory nature of the study.

#### Statistical methods

Categorical variables were compared using Chi-square tests and continuous variables using unpaired *t*-tests, with a significance level of *α* = 0.05. Missing data were minimal and handled by complete-case analysis. Continuous variables were reported as mean (SD) or median (IQR), and categorical variables as *n* (%).

The performance of the speech-based AI models was evaluated using standard classification metrics derived from the confusion matrix: true positives (TP), true negatives (TN), false positives (FP), and false negatives (FN). Accuracy was defined as (TP + TN)/(TP + TN + FP + FN), precision as TP/(TP + FP), recall (sensitivity) as TP/(TP + FN), and specificity as TN/(TN + FP). The F1 score was calculated as the harmonic mean of precision and recall. Unweighted average recall (UAR) was computed as the mean recall across classes. For multi-class classification, weighted F1, macro and balanced Matthew’s correlation coefficient (MCC) were used as a balanced measure of classification performance accounting for all elements of the confusion matrix, with values ranging from −1 (complete disagreement) to +1 (perfect classification). Macro and balanced MCC were reported for multi-class analyses where appropriate. Area under the receiver operating characteristic curve (ROC AUC) was used to assess overall discriminative performance across classification thresholds. No inferential statistical comparisons of model performance were performed because the analyses were exploratory and intended to evaluate feasibility rather than compare algorithms formally. Late fusion refers to a machine-learning approach in which predictions from separate models trained on different feature sets were combined at the decision level.

### Ethics

Ethical approval was obtained from the Ethics Committee and the Danish Data Protection Agency (case number 1-10-72-10-25) and was conducted in accordance with the ethical principles outlined in the Declaration of Helsinki and in compliance with applicable regulatory requirements. Written informed consent was obtained from all participants before enrolment. Data were pseudonymized and stored in GDPR-compliant AUH environments (REDCap).

## RESULTS

### Participants

A total of 63 participants were included: 41 cases and 22 controls (Fig. 1). Among eligible patients on haemodialysis, exclusions were due to declined participation, language barriers, sensory impairment, inability to provide consent, dementia, or dialysis duration <3 months. In the control group, four individuals declined participation, and one was excluded due to language barriers.

**Figure 1:**
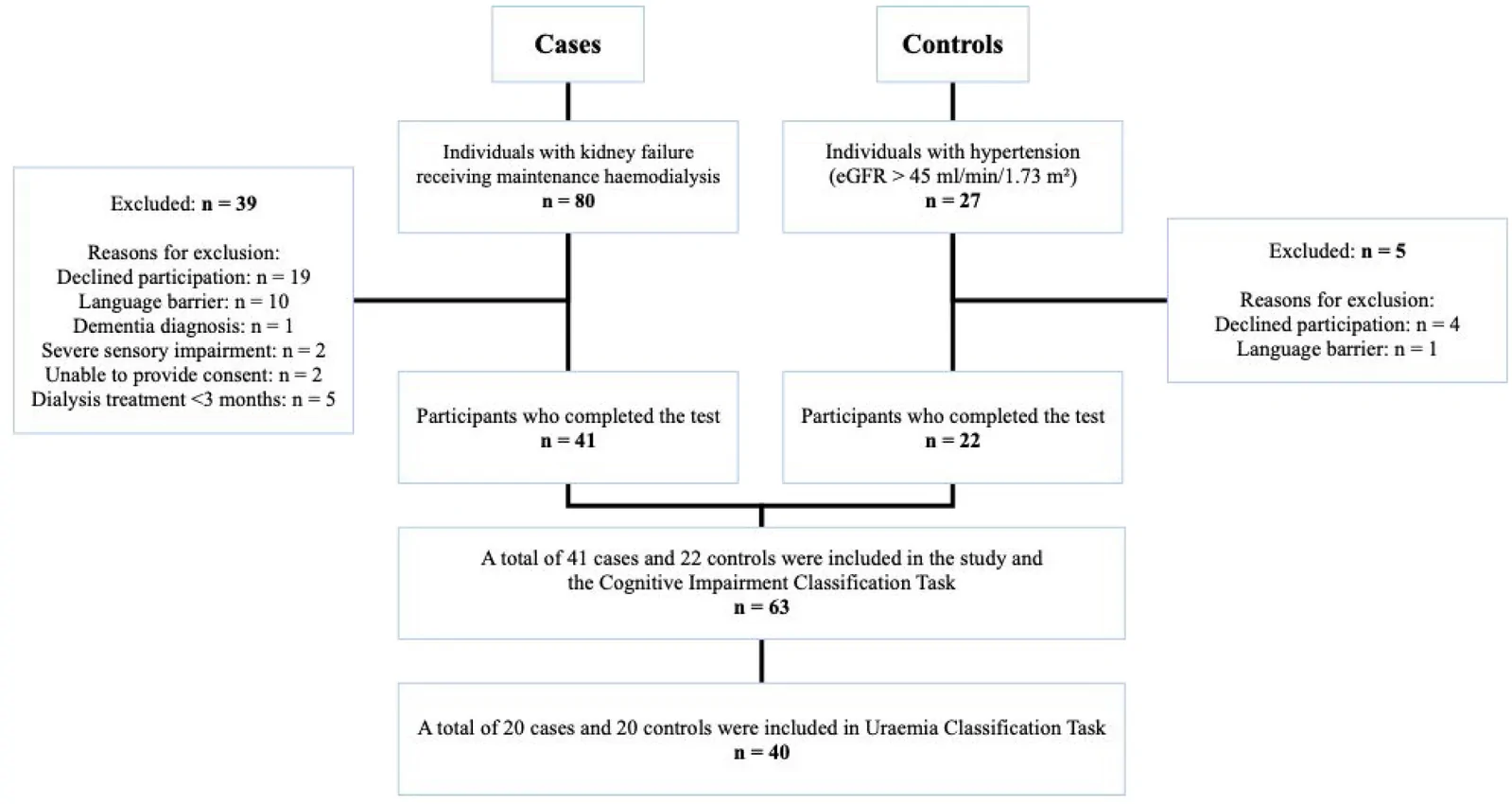
Flow diagram of participant inclusion and exclusion. Flow diagram illustrating the recruitment process for cases (patients receiving maintenance haemodialysis) and controls (patients from the hypertension clinic), including reasons for exclusion and final sample sizes included in the analysis.

MoCA-based CI classification task: among cases, the mean dialysis duration was 2.9 ± 3.5 years, and the mean weekly treatment time was 12.3 ± 2.8 h. Cases were older and less likely to be employed than controls, while being in a relationship was more prevalent among controls (Table 2). Median MoCA scores were lower in cases [23 (21–25)] than in controls [24.5 (23–26.6)].

**Table 2:** Participants characteristics.

	Cognitive impairment classification task				Uraemia classification task		
	Total *n = 63*	Case *n = 41*	Control *n = 22*	*P*-value	Case *n* = 20	Control *n* = 20	*P*-value
Age (year)							
Mean ± SD	64.7 ± 15.3	68.5 ± 13.6	57.6 ± 16.0	0.006	63.0 ± 13.7	57.5 ± 16.8	0.264
Sex *n* (%)							
Female	21 (33.3)	12 (29.3)	9 (40.9)	0.350	8 (40.0)	8 (40.0)	1.000
Education *n* (%)							
Primary	13 (20.6)	11 (26.8)	2 (9.1)		4 (20.0)	2 (10.0)	
Secondary	26 (41.3)	17 (41.5)	9 (40.9)		8 (40.0)	8 (40.0)	
Tertiary	24 (38.1)	13 (31.7)	11 (50.0)	0.179	8 (40.0)	10 (50.0)	0.641
Employment status *n* (%)							
Employed	20 (31.7)	7 (17.1)	13 (59.1)		11 (55.0)	8 (40.0)	
Unemployed	7 (11.1)	6 (14.6)	1 (4.5)		5 (25.0)	1 (5.0)	
Retired	36 (57.1)	28 (68.3)	8 (36.4)	0.003	4 (20.0)	11 (55.0)	0.041
Marital status *n* (%)							
In a relationship	35 (55.6)	18 (43.9)	17 (77.3)	0.011	9 (45.0)	15 (75.0)	0.053
Household composition *n* (%)							
Lives with other	36 (57.1)	20 (48.8)	16 (72.7)	0.067	10 (50.0)	14 (70.0)	0.197
MoCA-score							
Median (IQR)	24 (22;26)	23 (21;25)	24.5 (23;26.6)	0.007	24 (22.5;25.5)	24 (23:26.5)	0.303
MoCA-based CI categories							
Low risk (MoCA ≥26)	17 (27.0)	9 (22.0)	8 (36.3)		5 (25.0)	6 (30.0)	
Moderate risk (MoCA 23–25)	25 (39.7)	13 (31.7)	12 (54.6)		10 (50.0)	12 (60.0)	
High risk (MoCA ≤22)	21 (33.3)	19 (46.3)	2 (9.1)	0.011	5 (25.0)	2 (10.0)	0.459
Duration of dialysis (years)							
Mean ± SD		2.9 ± 3.5			2.2 ± 1.7		
Weekly dialysis time (hours)							
Mean ± SD		12.3 ± 2.8			11.5 ± 2.2		

SD = standard deviation, IQR = interquartile range.

Uraemia classification task: no statistically significant differences were observed between cases and controls with respect to age, sex, education level, marital status, household composition, or cognitive risk category. However, controls were more frequently retired than cases (55.0% vs. 20.0%; *P* = 0.041).

### Uraemia classification task

Model performance was evaluated across speech tasks (Table 1) using a denoised and balanced dataset with 20 cases and 20 controls.

Across tasks, acoustic and multimodal models showed comparable overall performance, with consistent advantages for the multimodal models in more complex tasks (Table 3). For speech task 5, the multimodal model reduced false positives (3 vs. 6), improved precision (0.68–0.80), and increased specificity (0.85 vs. 0.70), resulting in higher accuracy (0.72 vs. 0.60) and F1–score (0.69 vs. 0.67).

**Table 3:** Performance for the uraemia classification task.

		Confusion matrix				Metrics					
Speech task	Modality	TN	FP	FN	TP	Accuracy	F1-score	Precision	Recall	Specificity	UAR
5	Acoustics	14	6	7	13	0.68	0.67	0.68	0.65	0.70	0.68
	Linguistics	14	6	10	10	0.60	0.56	0.62	0.50	0.70	0.60
	Multi	17	3	8	12	0.72	0.69	0.80	0.60	0.85	0.72
6	Acoustics	16	4	5	15	0.78	0.77	0.79	0.75	0.80	0.78
	Linguistics	14	6	7	13	0.68	0.67	0.68	0.65	0.70	0.68
	Multi	15	5	4	16	0.78	0.78	0.76	0.80	0.75	0.78
5 + 6 [late fusion]	Acoustics	16	4	5	15	0.78	0.77	0.79	0.75	0.80	0.78
	Linguistics	15	5	10	10	0.62	0.57	0.67	0.50	0.75	0.62
	Multi	16	4	4	16	0.80	0.80	0.80	0.80	0.80	0.80

TN = true negatives, FP = false positives, FN = false negatives, TP = true positives, UAR = unweighted average recall.

For speech task 6, both models performed well. The acoustic model achieved an F1–score of 0.77 and accuracy of 0.78, while the multimodal model showed slightly higher F1–scores (0.78) and recall (0.80 vs. 0.75), indicating improved sensitivity. Late fusion further improved performance. The multimodal model achieved the highest and most balanced results across all metrics (0.80), indicating improved robustness when combining tasks and feature types.

### MoCA-based cognitive risk classification task

All 63 participants were included and categorized into three MoCA-based cognitive risk groups (Table 4).

**Table 4:** Participants' cognitive impairment categories.

MoCA-based CI risk category	Group	*n* (%)	Total (%)
Low	Control	8 (47)	17 (27)
	Case	9 (53)	
Moderate	Control	12 (48)	25 (40)
	Case	13 (52)	
High	Control	2 (9)	21 (33)
	Case	19 (91)	

Classification performance varied across speech tasks (Table 5). Among single–task models, speech task 2 showed the strongest and most consistent performance. For the high–risk category, it achieved a weighted F1–score of 0.73, ROC AUC of 0.70, and MCC of 0.41. Descriptive tasks (tasks 3–5) showed lower performance, with weighted F1–scores often below 0.60 and MCC values close to zero or negative in some categories. Across all tasks, specificity consistently exceeded sensitivity, particularly for low–risk participants, indicating more reliable identification of individuals with little or no MoCA-based CI. Sensitivity was the lowest for high–risk participants in descriptive tasks, (24%–29%), while fluency tasks showed more balanced performance, with sensitivity of 58%–62% and specificity of 76%–80%.

**Table 5:** Performance metrics for the cognitive impairment classification task.

Speech task	Model	CI risk category	Specificity	Sensitivity	UAR	Weighted F1	ROC AUC	MCC	bMCC	Macro bMCC
1	LGBMClassifier	Low	83	53	68	75	72	0.37	0.37	0.27
		Moderate	66	52	59	60	61	0.18	0.18	
		High	76	48	62	66	64	0.24	0.25	
2	BernoulliNB	Low	72	69	70	72	66	0.36	0.41	0.34
		Moderate	80	42	61	63	65	0.25	0.23	
		High	80	58	69	73	70	0.41	0.39	
3	XGBClassifier	Low	83	35	59	68	64	0.20	0.2	0.29
		Moderate	71	54	63	64	57	0.28	0.26	
		High	73	67	70	72	73	0.39	0.40	
4	RandomForestClassifier	Low	84	29	57	67	65	0.14	0.16	–
		Moderate	44	52	48	48	48	−0.03	−0.04	
		High	83	47	65	71	71	0.34	0.33	
5	LGBMClassifier	Low	74	29	52	62	52	0.03	0.04	0.20
		Moderate	68	48	58	60	58	0.17	0.17	
		High	79	62	70	73	68	0.45	0.41	
6	NearestCentroid	Low	67	41	54	62	53	0.07	0.09	0.14
		Moderate	66	60	63	64	63	0.27	0.26	
		High	81	24	52	59	50	0.05	0.06	

UAR = Unweighted Average Recall, MCC = macro and balanced Matthew’s correlation coefficient, ROC AUC = area under the receiver operating characteristic curve.

Late–fusion results are shown in Table 6. Combining all tasks resulted in moderate performance (weighted F1–scores up to 0.71, MCC up to 0.37). The best performance was observed in the two–task fusion (tasks 2 and 5), where the high–risk group achieved a weighted F1–score of 0.73, ROC AUC of 0.79, and MCC of 0.43. Fluency–only fusion (tasks 1–3) also performed well, with a high–risk F1–score of 0.70 and MCC of 0.35 alongside strong specificity for low–risk participants (83%). Overall, speech task 2 was the best-performing single task, and two–task fusion provided the most stable classification. Moderate–risk participants were consistently the most difficult to classify.

**Table 6:** Late fusion cognitive impairment classification task.

Tasks	Cognitive impairment category	Specificity	Sensitivity	UAR	Weighted F1	ROC AUC	MCC	bMCC	Macro bMCC
All tasks	Low	76	47	62	69	72	0.206	0.242	0.27
	Moderate	68	52	60	62	68	0.208	0.207	
	High	81	52	67	71	82	0.37	0.348	
Realistic set of tasks (diverse, still short): 2 and 5	Low	74	65	69	73	68	0.346	0.388	0.33
	Moderate	76	44	60	63	63	0.223	0.215	
	High	81	57	69	73	79	0.429	0.392	
Fluency only: 1, 2 and 3	Low	83	59	71	76	72	0.433	0.427	0.34
	Moderate	74	52	63	65	65	0.278	0.263	
	High	76	57	67	70	79	0.347	0.34	

## DISCUSSION

This proof–of–concept study suggests that speech–based AI can identify uraemia and MoCA-based cognitive risk in people with CKD. Multimodal models showed the strongest performance for uraemia classification, whereas verbal fluency tasks performed best for MoCA-based cognitive risk classification. Late–fusion approaches improved overall model stability. High–risk participants were identified with moderate accuracy, while moderate–risk participants were more difficult to classify. Across analyses, specificity consistently exceeded sensitivity.

### Challenges and clinical implications of speech–based AI

This study highlights both the potential and limitations of AI–based speech analysis for cognitive assessment in kidney failure. Model performance depended strongly on task type and feature representation. Notably, deep feature-based models in the uraemia classification task performed best on more spontaneous speech (e.g. picture description and narrative tasks), whereas MoCA-based cognitive risk classification based on more traditional features showed the highest performance for structured verbal fluency tasks. Phonemic fluency tasks yielded the most consistent and discriminative results for MoCA-based cognitive risk. However, this likely reflects partial circularity, as verbal fluency contributes directly to the MoCA score used to define cognitive risk categories. This introduces a degree of incorporation bias that may have inflated the apparent superiority of the verbal fluency tasks and should be considered when interpreting model performance. By contrast, more naturalistic speech tasks introduced greater variability and were harder to classify, although they retained some discriminative ability in binary settings separating low– and high–risk individuals. These findings illustrate a broader trade–off between ecological validity and classification performance [25]. Structured tasks may provide stronger and more reproducible signals but are less reflective of everyday communication, whereas spontaneous speech may better capture real–world cognitive function at the cost of reduced stability.

From a clinical perspective, speech–based AI offers a low–burden and scalable complement to existing cognitive assessments, particularly given the need for repeated monitoring in patients with CKD [3, 26, 27]. However, the moderate accuracy observed, especially for intermediate impairment, suggests that such tools are not yet suitable as stand–alone diagnostic instruments. These findings highlight the importance of rigorous validation and external testing before clinical deployment of AI-based clinical decision support tools.

If validated in larger cohorts, longitudinal speech analysis could provide insight into cognitive trajectories and their relationship to uraemia and dialysis–related factors [28, 29]. This may support earlier identification of cognitive decline and inform individualized care, including communication strategies, treatment planning, and follow–up intensity [3, 30].

### Complementary roles of uraemia and MoCA-based cognitive risk classification

The two classification tasks addressed complementary objectives: identifying speech patterns associated with uraemia and identifying MoCA-based cognitive risk. Together, they enabled differentiation between general uraemic effects and CI severity, strengthening the overall proof of concept.

### Strengths and limitations

This study has several strengths. To our knowledge, it is among the first to evaluate speech–based AI for both uraemia classification and MoCA-based cognitive risk detection in individuals receiving maintenance haemodialysis, and the first to assess cognition in Danish using both structured and spontaneous speech tasks. The inclusion of multiple tasks enabled comparison of task–specific performance, demonstrating complementary strengths across speech paradigms. The use of acoustic, linguistic, multimodal, and late–fusion approaches provided a comprehensive framework, while cross–validation enhanced robustness despite the exploratory design.

Several limitations should be considered. First, the relatively small sample size, particularly for the uraemia classification task, limits generalizability, increases uncertainty around performance estimates, raises the risk of overfitting given the high-dimensional feature space, and limits reliable quantification of uncertainty through confidence intervals. Furthermore, hyperparameter optimization, speech-task selection, and the evaluation of multiple algorithms, feature sets, speech tasks, and fusion approaches may have introduced optimistic bias into these internally derived performance estimates. Second, CI classification was based on MoCA, a screening tool rather than a diagnostic gold standard, and future studies should validate models against comprehensive neuropsychological assessment [20]. Third, although groups were partially balanced, cases and controls differed in clinical and demographic characteristics beyond kidney function, which may have influenced speech patterns and contributed to classification performance. Some model–identified features may therefore reflect general health differences rather than uraemia–specific effects. Fourth, speech production is inherently variable and may be influenced by factors such as fatigue, motivation, attention, mood, and treatment-related factors [31, 32]. Furthermore, differences in recording context may have influenced speech characteristics independently of cognitive status or uraemia. Consequently, the reported results should be considered exploratory, hypothesis-generating, and require confirmation in larger independent cohorts with external testing.

From a modelling perspective, the findings underscore the importance of rigorous preprocessing, balanced datasets, and robust validation strategies when developing speech–based AI models [13, 14, 33, 34]. Recordings were obtained in a real–world clinical environment rather than under highly standardized laboratory conditions; while this enhances ecological validity and reflects potential clinical use, it may also have introduced environmental noise and variability affecting model stability. Together, these considerations highlight the need for larger multicentre studies with repeated measurements, external validation, bias monitoring, and comprehensive neuropsychological assessment, as well as evaluation of feature stability, comparison of speech tasks, and integration into clinical workflows [13, 14, 33, 34].

## CONCLUSION

This proof-of-concept study suggests that speech-based AI may be able to identify uraemia and detect MoCA-based cognitive risk in individuals with kidney failure with moderate accuracy. Multimodal models and verbal fluency tasks showed the strongest classification performance, while late-fusion approaches improved overall stability. However, classification of moderate cognitive risk remained challenging. These findings suggest that speech-based AI may have potential as a scalable and non-invasive screening tool in nephrology. Further validation in larger, multicentre cohorts is required before its clinical utility can be established and to ensure generalizability.

## Supplementary Material

sfag316_Supplemental_Files

## Data Availability

The data underlying this article will be shared on reasonable request to the corresponding author.
