# Supplementary material for: AI and speech-based identification of cognitive impairment in maintenance haemodialysis—the ASPIRE study
Source: Clin Kidney J. 2026 Sep 11;19(10):sfag316. doi: 10.1093/ckj/sfag316 (PMC13653340; doi:10.1093/ckj/sfag316)
Supplement: Supplementary file 1 [file sfag316_supplemental_files.zip › Supplementary file 1.pdf]

Supplementary file 1

Performance for the Uraemia Classification Task - not denoised

Speech tasks	Modality	Confusion matrix				Metrics					
		TN	FP	FN	TP	Accuracy	F1-score	Precision	Sensitivity	Specificity	UAR
**Describe a picture**	Acoustic	11	9	4	34	0.78	0.84	0.79	0.89	0.55	0.72
	Linguistic	6	14	1	37	0.74	0.83	0.73	0.97	0.30	0.64
	Combined	11	9	1	36	0.83	0.88	0.80	0.97	0.55	0.76
**Balanced: Describe a picture**	Acoustic	14	6	5	15	0.72	0.73	0.71	0.75	0.70	0.72
	Linguistic	14	6	10	10	0.60	0.56	0.62	0.56	0.70	0.60
	Combined	16	4	7	13	0.72	0.70	0.76	0.65	0.80	0.73
**Cinderella story**	Acoustic	13	7	4	34	0.81	0.86	0.83	0.89	0.65	0.77
	Linguistic	8	12	1	37	0.78	0.85	0.76	0.97	0.40	0.69
	Combined	12	8	3	35	0.81	0.86	0.81	0.92	0.60	0.76
**Balanced: Cinderella story**	Acoustic	14	6	4	16	0.75	0.76	0.73	0.80	0.70	0.75
	Linguistic	14	6	7	13	0.68	0.67	0.68	0.65	0.70	0.68
	Combined	14	6	6	14	0.70	0.70	0.70	0.70	0.70	0.70
**Cheese sandwich**	Acoustic	12	8	4	34	0.79	0.85	0.81	0.89	0.60	0.75
	Linguistic	0	20	2	36	0.62	0.77	0.64	0.95	0.00	0.47
	Combined	*Irrelevant: linguistic at 0 TN*									
**Balanced: Cheese sandwich**	Acoustic	10	10	6	14	0.60	0.64	0.58	0.70	0.50	0.6
	Linguistic	13	7	12	8	0.53	0.46	0.53	0.40	0.65	0.52
	Combined	11	9	7	13	0.60	0.62	0.59	0.65	0.55	0.60
**Animals (1 minute)**	Acoustic	15	5	0	38	0.91	0.94	0.88	1.00	0.75	0.88
	Linguistic	0	20	2	36	0.62	0.77	0.64	0.95	0.00	0.47
	Combined	*Irrelevant: linguistic at 0 TN*									
**S-words (1 minute)**	Acoustic	15	5	4	34	0.84	0.88	0.87	0.89	0.75	0.82
	Linguistic	1	19	4	34	0.60	0.75	0.64	0.89	0.05	0.47
	Combined	*Irrelevant: linguistic at almost 0 TN*									

TN = true negative, FP = false positive, FN = false negative, TP = true positive
