# Supplementary material for: AI and speech-based identification of cognitive impairment in maintenance haemodialysis—the ASPIRE study
Source: Clin Kidney J. 2026 Sep 11;19(10):sfag316. doi: 10.1093/ckj/sfag316 (PMC13653340; doi:10.1093/ckj/sfag316)
Supplement: Supplementary file 1 [file sfag316_supplemental_files.zip › Supplementary file 2.pdf]

Supplementary file 2

Number	Model	Purpose
**1**	LGBMClassifier	A gradient boosting framework optimized for efficiency and performance, used to model complex non-linear relationships in high-dimensional feature spaces.
**2**	NearestCentroid	A distance-based classifier that assigns class labels based on proximity to class centroids, providing a simple and interpretable baseline
**3**	RandomForestClassifier	An ensemble learning method using multiple decision trees to improve classification performance and reduce overfitting
**4**	BernoulliNB	A probabilistic classifier based on the Naïve Bayes principle, particularly suited for binary or sparse linguistic features
**5**	XGBClassifier (Extreme Gradient Boosting)	A high-performance boosting algorithm capable of capturing complex feature interactions and non-linearities
